# Peer-Presented Versus Mental Health Service Provider–Presented Mental Health Outreach Programs for University Students: Randomized Controlled Trial

**DOI:** 10.2196/34168

**Published:** 2022-07-22

**Authors:** Laurianne Bastien, Bilun Naz Boke, Jessica Mettler, Stephanie Zito, Lina Di Genova, Vera Romano, Stephen P Lewis, Rob Whitley, Srividya N Iyer, Nancy L Heath

**Affiliations:** 1 Department of Educational and Counselling Psychology McGill University Montreal, QC Canada; 2 Student Services McGill University Montreal, QC Canada; 3 Department of Psychology University of Guelph Guelph, ON Canada; 4 Department of Psychiatry McGill University Montreal, QC Canada

**Keywords:** web-based mental health outreach, resilience building, university student, peer-presented, mental health service provider–presented, mental health, outreach, resilience, student, service provider, randomized controlled trial

## Abstract

**Background:**

University students are reporting concerning levels of mental health distress and challenges. University mental health service provider initiatives have been shown to be effective in supporting students’ mental health, but these services are often resource-intensive. Consequently, new approaches to service delivery, such as web-based and peer support initiatives, have emerged as cost-effective and efficient approaches to support university students. However, these approaches have not been sufficiently evaluated for effectiveness or acceptability in university student populations.

**Objective:**

Thus, the overarching goal of this study was to evaluate a mental health service provider–presented versus peer-presented web-based mental health resilience–building video outreach program against a wait-list comparison group.

**Methods:**

Participants were 217 undergraduate students (mean age 20.44, SD 1.98 years; 171/217, 78.8% women) who were randomly assigned to one of the intervention groups (mental health service provider–presented: 69/217, 31.8%; peer-presented: 73/217, 33.6%) or the wait-list comparison group (75/217, 34.6%). Participants in the intervention groups were asked to watch 3 brief skill-building videos addressing strategies for building mental health resilience, whereas the comparison group was wait-listed. The mental health service provider–presented and peer-presented video series were identical in content, with presenters using a script to ensure consistency across delivery methods, but the videos differed in that they were either presented by mental health service providers or university students (peers). All participants were asked to complete web-based self-report measures of stress, coping self-efficacy, social support, social connectedness, mindfulness, and quality of life at baseline (time 1), 6 weeks later (time 2, after the intervention), and 1-month follow-up (time 3).

**Results:**

Results from a series of 2-way ANOVAs found no significant differences in outcomes among any of the 3 groups. Surprisingly, a main effect of time revealed that all students improved on several well-being outcomes. In addition, results for program satisfaction revealed that both the mental health service provider–presented and peer-presented programs were rated very highly and at comparable levels.

**Conclusions:**

Thus, findings suggest that a web-based mental health resilience–building video outreach program may be acceptable for university students regardless of it being mental health service provider–presented or peer-presented. Furthermore, the overall increases in well-being across groups, which coincided with the onset and early weeks of the COVID-19 pandemic, suggest an unexpected pattern of response among university students to the early period of the pandemic. Limitations and barriers as well as research implications are discussed.

**Trial Registration:**

ClinicalTrials.gov NCT05454592; https://clinicaltrials.gov/ct2/show/NCT05454592

## Introduction

### Background

Over the past decade, mental health difficulties among university students have become a significant concern, with reports of 20% of students experiencing clinical depression and approximately 59% reporting experiencing above-average to tremendous levels of stress over the previous 12 months [1]. Although the traditional mental health service provider support offered through universities has been shown to be effective in increasing well-being among students, it is often costly and resource-intensive and can incur lengthy wait times because of the overwhelming demand [2-4]. Web-based peer initiatives have been suggested as cost-effective and efficient approaches to provide additional support and build capacity for mental health resilience among university students, but studies report mixed findings on the effectiveness of these approaches and a need for an evidence-based skill-building focus in these types of interventions [5-8]. Thus, the main objective of this study was to explore the acceptability and effectiveness of a web-based mental health resilience–building program as well as to evaluate differences between mental health service provider–presented and peer-presented variations of the program.

Evidence shows that university students are experiencing heightened levels of mental health distress. The National College Health Assessment survey across Canadian campuses with 55,284 student respondents revealed that 69% of students reported feeling overwhelming anxiety and 88% felt overwhelmed within the last year [1]. In addition to these heightened levels of stress, the developmental period of emerging adulthood, which is a theoretically and empirically distinct developmental period that takes place between adolescence and adulthood (ie, between the ages of 18 and 29 years), has been associated with a peak in unhealthy coping behaviors such as alcohol and drug abuse [9,10]. Characteristics of this developmental period include instability (ie, feeling like aspects of one’s life such as relationships and work are unstable or easily subject to change) and feeling *in-between* (ie, feeling that they are not an adolescent anymore but not yet feeling like an adult), which have been found to have important mental health implications such as feelings of depression and anxiety [11,12]. As such, there is a clear need to provide university students with appropriate and effective support for building resilience and managing stress [13].

Leading organizations in health promotion have indicated a need for preventative programs aimed at enhancing mental health resilience. Specifically, the World Health Organization has identified increasing self-management and self-care ability through skill development as a core area to be addressed in efforts to enhance the mental health of emerging adults [14]. Furthermore, they note that increasing self-management and self-care would, in turn, result in concomitant decreases in demand for more intensive therapeutic interventions.

### Current Mental Health Support

Several evidence-based self-care and stress management strategies (eg, mindfulness strategies, progressive muscle relaxation, diaphragmatic breathing, and emotion regulation strategies) have been shown to promote resilience through effectively reducing stress and increasing well-being in emerging adults [15]. Students will often access these strategies through professional counseling support, which has been linked to a significant decrease in distress symptoms and improvement in academic performance [16,17].

Although many of these strategies and programs can be effective in supporting university students, they are often presented in individualized therapy and counseling sessions (eg, dialectical behavioral therapy and cognitive behavioral therapy), and these programs often operate at a significant financial cost [2,3]. Owing to heightened demands, this reduces the feasibility and access to such programs for all university students experiencing mental health distress (absent of mental illness) in university environments. These findings highlight the potential need for increasing access to these evidence-based skills to improve university students’ ability to manage stress and enhance their coping during the challenging developmental period of emerging adulthood.

### New Approaches for Additional Support

Commensurate with the aforementioned strategies, new cost-effective approaches to service delivery are now being explored, including web-based mental health support. Web-based support takes advantage of the *new digital age*, in which increasing numbers of people (especially emerging adults) normatively obtain information, connection, and support via mobile phones, tablets, and home computers [18]. Web-based approaches also provide flexibility as they are self-paced (ie, fit into students’ busy schedules as they can be used at any preferred time) [19,20]. Furthermore, another benefit of web-based mental health resources is the anonymity provided to students who may be reluctant to seek support because of stigma related to mental health [19]. Thus, the development of web-based mental health resources can provide improved access to evidence-based support to build mental health resilience on campuses, but new efforts must be based on solid research and scientific evidence [21]. Of note, an emerging body of research demonstrates that web-based mental health support can provide effective, efficient, and cost-effective support for individuals experiencing mental health distress (absent of mental illness), but there is still a need for innovation and evaluation to optimize student-oriented support [21,22]. Specifically, enhancing the relatability of the content or delivery of web-based mental health resources has been suggested to address low engagement or use of resources found in numerous studies of existing web-based support [23,24].

Peer support initiatives have also been identified as a promising approach for providing universal mental health resilience support. These initiatives require fewer professional resources and have been found to promote empowerment among individuals facing mental health challenges [7]. Peer support initiatives serve to fill gaps in official service provision and provide students with informal, peer-focused support with an emphasis on shared experience as opposed to psychopathology [25]. These initiatives allow peers to provide assistance to others drawing on their own lived experience of mental health challenges and to help others in their recovery journey [26]. They can also help decrease stigma and increase help-seeking behaviors through the sharing of information by those with similar experiences [7]. Indeed, evidence shows that, when students experience mental health difficulties, they tend to first turn to their peers for support in discussing these types of challenges [27,28]. Accordingly, peer support and web-based mental health outreach may be interesting to examine as approaches to provide access to evidence-based resilience-building strategies for university students. Although web-based mental health resources and peer support approaches are gaining popularity, many are not evidence-based, have privacy and confidentiality concerns, or have not been sufficiently evaluated for effectiveness or acceptability in university student populations [5,29,30]. Moreover, further research is needed to understand whether there are differences in the acceptability, satisfaction with, and effectiveness of a web-based mental health program as a function of whether the program is delivered by a mental health service provider or peer.

### This Study

Drawing on the aforementioned literature, the overarching goal of this study was to evaluate a mental health service provider–presented versus peer-presented mental health resilience skill–building web-based video outreach program against a wait-list comparison group. To our knowledge, this is the first study to examine acceptability of and satisfaction with a mental health service provider–presented versus peer-presented universal resilience-building web-based program in a sample of university students.

#### Objective 1

The first objective was to evaluate the acceptability of and satisfaction with a mental health service provider–presented versus peer-presented web-based skill-building video outreach program for university students. Given the exploratory nature of objective 1 of this study, no specific hypotheses were made.

#### Objective 2

The second objective was to compare group differences between a mental health service provider–presented versus peer-presented web-based skill-building video outreach program and a wait-list comparison group in terms of well-being outcomes over 10 weeks. It was hypothesized that the intervention groups (mental health service provider–presented and peer-presented) would demonstrate a greater increase in well-being outcomes (effectiveness) compared with the wait-list comparison group.

## Methods

### Participant Eligibility

Participants were eligible for the study if they were aged between 18 and 29 years given the unique stressors associated with the developmental period of emerging adulthood (age 18-29 years [11]). Furthermore, participants were required to have access to the internet (at least weekly) as the study was completed entirely on the web.

### Program Development and Description

The web-based mental health outreach program for university students was developed using an approach inspired by principles of the Participatory Action Research model, defined as “a partnership among equals with complementary knowledge and expertise” in which three key elements are collaboration, education, and action [31,32]. Consistent with the Participatory Action Research model, the program was developed using the expert knowledge of evidence-based strategies and best-practice applications of a multidisciplinary team of researchers (n*=*4), student service users with lived experience of mental health challenges (approximately 8-10 core team members who were consistently involved throughout the study and 15 team members whose participation in the project was fluid), mental health service providers (n*=*3), and decision makers (n*=*2). All stakeholders were actively involved throughout the project and consulted for project-related decisions (eg, study design and conceptualization and program development and dissemination). The multidisciplinary team met twice per month on average and reached consensus on all aspects of the program after lengthy discussion. In addition, meeting minutes were sent following each meeting, and all members were encouraged to reply via email directly to the project coordinator if they felt that (1) there was any discrepancy between the meeting minutes and what had been agreed upon, (2) there was any missing information, and (3) they had any questions or additional feedback they would like to provide regarding the decisions that were made. Alternatively, team members could edit the meeting minutes web-based document directly if they were more comfortable that way.

The web-based outreach program focused on four key areas of mental health resilience–building identified by the multistakeholder team’s expertise and review of the literature: dealing with stress, decreasing self-criticism, improving self-care and help-seeking behaviors, and enhancing social connections and social support [33-37]. Using videos, web-based infographics, guided audio recordings, and podcasts, students were provided with clear descriptions of each area of mental health resilience as well as a variety of evidence-based strategies (Table 1) specifically targeting one or more of these areas. The program was hosted entirely on the web, and students were encouraged to access the materials most relevant to their needs. A first video was sent to the students describing the web-based program, its overall focus, and how to access the skill-building strategies on the website’s resource library. At a 2-week interval, 2 subsequent videos were sent to (1) help students with problem solving for common challenges to strategy practice and (2) maintain long-term strategy practice habits. To assess differences in terms of preference for deliverer, two series of videos were created: one in which the deliverers were mental health service providers and one in which they were undergraduate students (ie, peers). Presenters were introduced at the beginning of the videos identifying themselves as a university student or mental health service provider working with university students. Their names and titles (mental health service provider or university student) also appeared at the bottom of the screen in the introduction. Attention was paid to ensure continuity of presenter characteristics with representation in terms of gender and race or ethnicity in both the mental health service provider–presented and peer-presented videos. The videos were identical in content in that the presenters used a script to ensure consistency across delivery methods; however, the videos differed in that they were either mental health service provider–presented or peer-presented. Students in the intervention groups (peer-presented and mental health service provider–presented) had access to the resource library throughout the study (ie, 9 weeks). The resource library was a website where students could select a strategy based on the area of resilience building that they wanted to work on, and they would be directed to an infographic or an audio recording to walk them through the different strategies. Students could access the resource library anywhere at any time as these resources were completely self-paced.

**Table 1:** Strategies presented in the resource library with the relevant key areas for resilience building that they address.

Strategy	Key areas for resilience building that the strategy addresses			
	Dealing with stress	Decreasing self-criticism	Improving self-care and help seeking	Enhancing social connections and support
Mindfulness on the go (infographic)	✓^a^	✓	✓	✓
Thought challenge (infographic)	✓	✓	✓	✓
Sitting meditation (audio recording)	✓	✓	✓	✓
Self-compassion meditation (audio recording)	✓	✓	✓	✓
Acceptance affirmation (infographic)	✓	✓	✓	✓
Body scan (audio recording)	✓	✓	✓	
Three good things (infographic)	✓	✓	✓	
Dealing with breakups (infographic and podcast)	✓		✓	✓
Calming breath (audio recording)	✓		✓	✓
Social network in university (infographic and podcast)	✓		✓	✓
Beyond time management (infographic)	✓		✓	✓
Physical well-being (infographic)	✓		✓	
Riding the wave (infographic)	✓	✓		
Self-care assessment (infographic)	✓		✓	
Sleep hygiene (infographic)	✓		✓	
Smart nutrition (infographic)	✓		✓	
Yoga nidra (infographic and audio recording)	✓		✓	
Progressive muscle relaxation (audio recording)	✓			
Financial wellness (infographic)	✓			

^a^✓: The strategy is presented in the resource library under the relevant area of resilience building.

### Procedure

#### Overview

Participants were recruited using a study flyer distributed to students in person on campus and on the web through email listservs and social media platforms and from an existing database of university students who had participated in previous studies and agreed to be followed up with. Given the self-paced nature of the program, a staggered recruitment approach was used wherein new participants completed the web-based baseline questionnaires between February 2020 and early March 2020.

Participants were randomly assigned to one of the three groups (mental health service provider–presented, peer-presented, or a wait-list comparison group) while counterbalancing the three groups based on gender and preference for seeking help from mental health service providers or peers (ie, using results from the General Help-Seeking Questionnaire [GHSQ]) [38]. Specifically, to minimize the risk of imbalance of confounding factors (ie, gender and help-seeking preference) within the different groups, a minimization-based approach was used [39]. Minimization has been recommended for smaller trials (<1000) where specific participant factors (eg, gender) may influence the outcome [40]. Thus, participants were allocated to one of the three groups (mental health service provider–presented, peer-presented, or comparison) based on their scores on (1) formal and (2) informal help-seeking items on the GHSQ (classified as high, medium, or low scores) as well as (3) gender (classified as male, female, or nonbinary). Thus, the participants were randomized on an ongoing basis as they were enrolled in the study. As per minimization, the first participant is allocated to a group completely at random, but subsequent participants’ group allocation depends on the characteristics of the participants already enrolled [39]. For example, if the next participant to be allocated had a profile with the following characteristics—female with high formal and medium informal help-seeking preferences—and there were already 5 participants with these characteristics in the mental health service provider–presented group but there were 6 participants in the peer-presented group and 6 participants in the comparison group, then this participant was allocated to the mental health service provider–presented group. The goal was for each allocation to minimize the imbalance across groups based on multiple factors.

**Figure 1:**
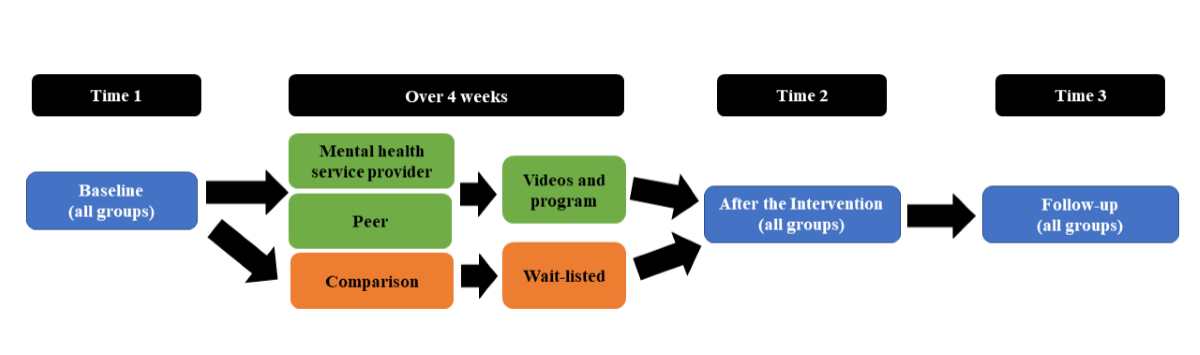
Project timeline for the stress and coping web-based outreach program.

A week after the baseline questionnaire was sent, participants in the intervention groups received either the mental health service provider–presented video or the peer-presented video (video 1) depending on which group they were randomly assigned to, as well as a link for access to the resource library. The next 2 videos were sent 2 weeks apart. Participants were encouraged to access the resource library over the duration of the program and were reminded with each video link sent. All participants then received postintervention (time 2) and follow-up (time 3) measures 6 and 10 weeks following baseline completion, respectively (see Figure 1 for the project timeline). Participants in the wait-list comparison group were only asked to complete evaluation measures at the 3 time points (they only received the videos and resource library at the end of the study).

Following completion of the study, the students received an email with a personalized profile indicating their individual scores on various measures, and all participants received full access to the program resources (videos and resource library). The participants were compensated CAD $10 (US $ 7.73) for each survey completed for a total of CAD $30 (US $23.18) and were also entered in a raffle for a 1 in 4 chance to win CAD $50 (US $38.64).

#### COVID-19 Context

In March 2020, when most students received the web-based mental health outreach program, a state of emergency was declared in the city in which this study was conducted. This resulted in the closure of all recreational centers, public parks and playgrounds, public libraries, bars, restaurants, movie theaters, concert venues, and places of worship as well as the banning of public gatherings. As per public health guidelines, all residents were recommended to stay home unless purchasing necessities (eg, food and supplies), for medical need, for essential work travel, or for 1 form of exercise per day. Strict social distancing guidelines prohibited in-person gatherings, and travel restrictions were implemented. In addition, the university in which this study took place was closed for a period of 2 weeks following the students’ reading week (week off for spring break). There was a transition to web-based learning, the university allowed for flexibility for final assignments (students could be provided with extensions, and some final assignments were removed), and students were provided with a pass or fail option rather than a final grade. This was a period of drastic changes and increased social isolation for university students [41]. The data for this study were collected at three time points: before the pandemic (time 1; February 2020), pandemic onset (time 2; March 2020-April 2020), and early pandemic (time 3; April 2020-May 2020).

### Measures

#### General Help-Seeking

The GHSQ [38] is a 10-item measure of formal and informal help seeking and uses the following prompt: “If you were having a personal or emotional problem, how likely is it that you would seek help from the following people?” The GHSQ was adapted to include help from classmates, academic advisors, residence supports, professors, research supervisors, and peer support organizations. Scores on willingness to seek support from peers and web-based resources (ie, informal) as well as willingness to seek support from professionals (ie, formal) were examined to randomize participants across program groups (mental health service provider–presented group, peer-presented group, and wait-list comparison group). The GHSQ has good reliability (Cronbach α=.91) and good construct validity [38]. In this study, the GHSQ had good internal consistency, with a Cronbach α at time 1 of .70.

#### Training Satisfaction

The *Response to Training* is a researcher-developed measure assessing participants’ satisfaction with the program content and delivery. The questions were delivered according to the three levels of the New World Kirkpatrick Model [42] as follows: (1) student viewers’ response (ie, satisfaction, engagement, and relevance), (2) learning (ie, knowledge, skills, attitude, confidence, and commitment), and (3) use of skills (ie, willingness to use and frequency of use). All items were scored on a 4- to 6-point Likert scale where higher scores represented a better response to training. Sample items include “I would recommend the program to other university students” or “I am planning to use the program strategies in the future.”

#### Perceived Stress

The *Perceived Stress Scale* (PSS) [43] is a widely used self-report measure of individuals’ perception of stress. This measure contains 10 items in which participants indicate their experience of stress on a 5-point Likert scale (0=*never* to 4=*very often*). The participants were asked to think about their lives over the previous month for baseline (consistent with the original scale) and over the previous 3 weeks (to assess the appropriate period after program use) for postintervention and follow-up measurements. Sample items include “In the past month/3 weeks, how often have you felt difficulties were piling up so high that you could not overcome them?” Ratings were averaged across items such that higher scores represented greater perceived stress. The PSS has good reliability (Cronbach α=.89), construct validity, and predictive validity with reports of psychological and physical symptoms [44,45]. In this study, the PSS had good internal consistency, with a Cronbach α of .86, .82, and .85 at time points 1, 2, and 3, respectively.

#### Coping Self-efficacy

The *Coping Self-Efficacy Scale* (CSE) [46] is a measure of one’s confidence in effectively engaging in coping behaviors in the face of challenges. This measure contains 26 items in which participants indicate confidence in their coping strategies when it comes to handling challenges and stressors on an 11-point Likert scale (0=*cannot do at all* to 10=*certain can do*). The participants were asked to think about their lives over the previous month for baseline (consistent with the original scale) and over the previous 3 weeks (to assess the appropriate period after program use) for postintervention and follow-up measurements. The CSE states that “When things aren’t going well for you, or when you’re having problems how confident or certain are you that you can do the following” and includes statements such as “find solutions to your most difficult problems” and “see things from the other person’s point of view during a heated argument.” Higher scores on the CSE represent higher coping self-efficacy. The CSE has good internal consistency (Cronbach α=.91) and test-retest reliability [46]. In this study, the CSE had good internal consistency, with a Cronbach α at time points 1, 2, and 3 of .92, .93, and .94, respectively.

#### Social Support

The *Multidimensional Scale of Perceived Social Support* (MSPSS) [47] is a 12-item self-report questionnaire developed to assess the subjective perception of social support adequacy from family, friends, and significant others. Items are rated on a 7-point Likert scale (1=*strongly disagree* to 7=*strongly agree*). Participants were asked to think about their lives over the previous month for baseline (consistent with the original scale) and over the previous 3 weeks (to assess the appropriate period after program use) for postintervention and follow-up measurements. Sample items include “There is a special person who is around when I am in need” and “My family really tries to help me.” Higher scores on the MSPSS represent higher perception of social support. The MSPSS has good reliability (Cronbach α ranging from .81 to .98) and good convergent and construct validity [48]. In this study, the MSPSS had good internal consistency, with a Cronbach α at time points 1, 2, and 3 of .89, .91, and .92, respectively.

#### Social Connectedness

The *Social Connectedness Scale-Revised* (SCS-R) [49] is a 20-item self-report questionnaire that assesses emotional distance of the self from both friends and society along with maintaining a sense of closeness. Items are rated on a 6-point Likert scale (1=*strongly disagree* to 6=*strongly agree*). Participants were asked to think about their lives over the previous month for baseline (consistent with the original scale) and over the previous 3 weeks (to assess the appropriate period after program use) for postintervention and follow-up measurements. Sample items include “I feel distant from people” and “I am able to relate to my peers.” Higher scores on the SCS-R represent higher perception of social connectedness. The SCS-R has good internal reliability (Cronbach α=.92) and good convergent and discriminant validity [49]. In this study, the SCS-R had good internal consistency, with a Cronbach α at time points 1, 2, and 3 of .90, .89, and .91, respectively.

#### Mindfulness

The *Mindful Attention Awareness Scale* (MAAS) [50] measures individuals’ dispositional mindfulness (ie, general tendency to be mindful) by assessing the frequency of mindful states over time. The MAAS consists of 15 items asking participants to report the frequency with which they have certain experiences on a 6-point scale (1=*almost always* to 6=*almost never*). Participants were asked to think about their lives over the previous month for baseline (consistent with the original scale) and over the previous 3 weeks (to assess the appropriate period after program use) for postintervention and follow-up measurements. Sample items include descriptions of experiences such as “I find myself preoccupied with the future or the past” and “I find myself doing things without paying attention.” Scores for this measure are such that higher scores indicate higher levels of mindfulness. The MAAS has demonstrated strong internal consistency (Cronbach α=.89) as well as high test-retest reliability and convergent and discriminant validity [51]. In this study, the MAAS had good internal consistency, with a Cronbach α at time points 1, 2, and 3 of .80, .79, and .91, respectively.

#### Quality of Life

The *World Health Organization Quality of Life Brief* questionnaire (WHOQOL-BREF) [52] is a 26-item measure assessing individuals’ perception of their life quality within the following domains: physical health, psychological health, social relationships, and their environment. Participants are asked to rate items related to their experience of their own quality of life (QoL) on a 5-point Likert scale (1=*not at all* to 5=*extreme amount*). Participants were asked to think about their lives over the previous month for baseline (consistent with the original scale) and over the previous 3 weeks (to assess the appropriate period after program use) for postintervention and follow-up measurements. Sample items include “To what extent do you feel that physical pain prevents you from doing what you need to do?” and “How satisfied are you with the conditions of your living place?” The WHOQOL-BREF shows decent reliability (Cronbach α values for physical health, psychological health, social relationships, and environmental health were .65, .77, .52, and .79, respectively) and good internal consistency [53]. In this study, the WHOQOL-BREF had acceptable internal consistency. Specifically, the Cronbach α for the 4 domains ranged from .49 to .79 at time points 1, 2, and 3.

### Data Analysis

All data were analyzed using SPSS (version 26; IBM Corporation). The data were checked for patterns of missingness, univariate and multivariate outliers, and violations of assumptions before running the main analyses. A series of chi-square tests were used to test the first objective, which was to compare group differences on the web-based outreach program’s acceptability between the types of deliverers (mental health service provider vs peer). A series of 2-way mixed ANOVAs were used to test the second objective, which was to compare group differences between a mental health service provider–presented and peer-presented web-based skill-building video outreach program and a wait-list comparison group in terms of well-being outcomes. To account for multiple pairwise comparisons (a total of three: mental health service provider, peer, and comparison) throughout the data analysis, the *P* value cutoff for statistical significance was set at .02 (.05 divided by 3) as per the Bonferroni correction.

### Ethics Approval

This study was approved by the McGill University Review Ethics Board (19-11-031).

## Results

### Participants

On the basis of data analysis requirements, a priori power analyses conducted with G*Power (version 3.1.9.6; Faul) with a medium effect size [54-56] and a power of 0.80 suggested minimum sample sizes of 186 [57]. Therefore, to account for attrition, a total of 294 undergraduate students were recruited (mean age 20.50, SD 2.35 years; 171/217, 78.8% women). However, of those 294 students who consented to participate, following data cleaning and participant withdrawal or dropout, the total sample of participants who completed all 3 time points was 217 (73.8%) undergraduate students (mean age 20.44, SD 1.98 years). Of this final sample of 217 undergraduate students, 171 (78.8%) self-identified as women, 42 (19.4%) self-identified as men, and 4 (1.8%) self-identified as nonbinary. The participants self-identified as White (132/217, 60.8%), Asian (54/217, 24.9%), Hispanic or Latino (11/217, 5.1%), African American or Black (10/217, 4.6%), Middle Eastern (7/217, 3.2%), and Indigenous (3/217, 1.4%) and, of these participants, 35.9% (78/217) reported being international students (the proportion of international students is comparable with the proportion at the university, which is approximately 30%) [58]. The participants were enrolled in different academic faculties, including Arts (88/217, 40.6%), Science (39/217, 18%), Agricultural and Environmental Studies (21/217, 9.7%), Engineering (20/217, 9.2%), Education (19/217, 8.8%), Management (10/217, 4.6%), and others (20/217, 9.2%). Of this sample, 74.7% (162/217) of students reported having experienced stress or mental health or well-being difficulties at a level that interfered with their ability to engage in the activities of everyday life (eg, school, work, relationships, and health-promoting behaviors) within the previous year. Furthermore, 25.3% (55/217) of the participants reported currently accessing mental health services such as counseling or therapy.

### Data Cleaning

**Figure 2:**
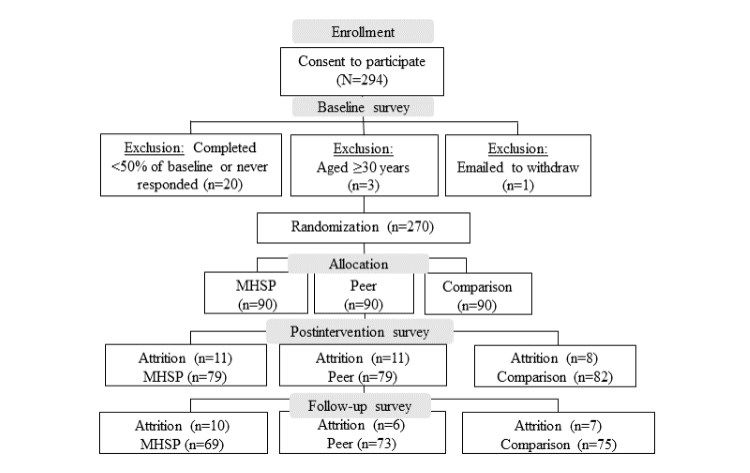
Attrition and exclusion of participants. MHSP: mental health service provider.

A total of 294 individuals consented to participate in this study (230/294, 78.2% women; mean age 20.50, SD 2.35 years). Of this total sample, 0.3% (1/294) of the participants withdrew before being randomized to an intervention group providing a lack of time to participate in the study as a reasoning for the withdrawal, and 1% (3/294) were excluded as they were aged ≥30 years, which was an a priori exclusion criterion for the trial. In addition, 18% (53/294) of the participants were lost because of attrition (see Figure 2 for further details on the study sample).

Before running primary analyses, a missing values analysis was conducted and revealed that data were missing completely at random given that <5% of data points were missing per variable [59]. To preserve the sample size, the Expectation Maximization imputation method was used, where missing values were imputed within each subscale of measures in the mental health service provider–presented, peer-presented, and wait-list comparison groups separately to maximize prediction accuracy. The data were then screened for potential univariate outliers within each of the dependent variables. Cases 3 SDs above or below the mean were identified as potential outliers. A total of 14 potential univariate outliers were identified and Winsorized to a score with a 1-unit difference from the next most extreme score within each variable to maintain rank order. No multivariate outliers or violations of normality were found within any of the 3 groups. All assumptions for the 2-way mixed ANOVAs were met satisfactorily.

### Preliminary Analyses

To determine whether randomization effectively balanced the groups across well-being outcomes, multiple 1-way ANOVAs examining group (mental health service provider–presented, peer-presented, and wait-list comparison) differences in baseline stress, coping self-efficacy, social support, social connectedness, mindfulness, and QoL were conducted. Results from the 1-way ANOVAs revealed no significant differences, indicating that none of the groups differed on any of the well-being outcomes at baseline and that the groups were comparable. The means and SDs for the well-being outcomes of each group are shown in Table 2. In addition, demographics were comparable across groups. Gender was accounted for to ensure distribution across groups during randomization, and age was comparable across groups (mental health service provider–presented: mean age 20.40, SD 1.86 years; peer-presented: mean age 20.22, SD 1.96 years; wait-list comparison: mean age 20.58, SD 2.06 years).

**Table 2:** Preliminary 1-way ANOVAs for group differences (mental health service provider–presented, peer-presented, and wait-list comparison) at baseline.

Variable	Mental health service provider–presented, mean (SD)	Peer-presented, mean (SD)	Wait-list comparison, mean (SD)	ANOVA results	
				*F* test (*df*)	*P* value
Stress	21.37 (5.76)	22.58 (6.99)	22.41 (5.77)	0.84 (2,214)	.43
Coping self-efficacy	134.23 (28.97)	137.1 (39.24)	134.7 (38.37)	0.20 (2,214)	.82
Social support	5.19 (0.96)	5.22 (1.01)	5.21 (1.07)	0.07 (2,214)	.93
Social connectedness	75.71 (15.97)	80.83 (18.06)	78.79 (18.08)	2.14 (2,214)	.12
Mindfulness	3.57 (0.81)	3.64 (0.83)	3.64 (0.74)	0.37 (2,213)	.70
QoL^a^ (physical health)	100.89 (15.95)	102.00 (16.35)	102.08 (16.35)	0.21 (2,212)	.82
QoL (psychological health)	74.24 (15.57)	74.58 (16.72)	72.37 (17.29)	0.30 (2,214)	.75
QoL (social relationships)	38.83 (9.66)	39.56 (9.77)	40.59 (9.34)	0.84 (2,214)	.43
QoL (environment)	116.12 (17.18)	116.8 (21.61)	115.5 (18.61)	0.10 (2,211)	.90

^a^QoL: quality of life.

### Main Analyses

#### Objective 1

The first objective sought to evaluate the acceptability and satisfaction of a mental health service provider–presented versus peer-presented web-based skill-building video outreach program for university students. A series of chi-square tests were conducted using the training satisfaction survey at time 2 (Table 3). Results from the chi-square tests revealed that there was no significant difference between the mental health service provider–presented and peer-presented acceptability of the program on any of the training satisfaction items selected to represent each level of the Kirkpatrick model (student viewers’ response, learning, and use of skills). Overall, the results of the training satisfaction survey demonstrate that most students were satisfied with the program (Table 3). For example, 81% (58/72) of the students in the mental health service provider–presented group and 91% (69/76) of the students in the peer-presented group said that they were planning to use the program strategies *sometimes* to *frequently*. In addition, 96% (69/72) of the students in the mental health service provider–presented group and 99% (75/76) of the students in the peer-presented group said that they *somewhat agreed* to *strongly agreed* that they would recommend the program strategies to other university students. By time 3, 65% (51/79) of the students in the mental health service provider–presented group and 78% (63/81) of the students in the peer-presented group reported having used the program strategies to cope with COVID-19 stress.

**Table 3:** Training satisfaction by group.

Survey item and response options			Mental health service provider–presented, n (%)		Peer-presented, n (%)		Chi-square (*df*)		*P* value		Cramer *V*		*P* value
**I used the SCOOP^a^ strategies**							1.8 (2)		.40		0.112		.22
	Never to rarely	27 (38)^b^		22 (29)^c^									
	Sometimes	40 (56)^b^		45 (59)^c^									
	Frequently	5 (7)^b^		9 (12)^c^									
**I am planning to use the SCOOP strategies in the future.**							4.0 (2)		.14		0.164		.14
	Never to rarely	14 (19)^b^		7 (9)^c^									
	Sometimes	36 (50)^b^		48 (63)^c^									
	Frequently	22 (31)^b^		21 (28)^c^									
**I would recommend the SCOOP strategies to other university students.**							1.1 (2)		.59		0.084		.59
	Strongly disagree to somewhat agree	25 (35)^b^		24 (32)^c^									
	Agree	34 (47)^b^		33 (43)^c^									
	Strongly agree	13 (18)^b^		19 (25)^c^									
**Video 1—after watching this video, I feel I learned...**							2.8 (2)		.24		0.139		.24
	Nothing to a small amount	25 (35)^d^		23 (30)^c^									
	A medium amount	33 (46)^d^		30 (39)^c^									
	A lot	13 (18)^d^		23 (30)^c^									
**Video 2—after watching this video, I feel I learned...**							2.3 (2)		.32		0.129		.32
	Nothing to a small amount	29 (42)^e^		21 (30)^f^									
	A medium amount	33 (48)^e^		39 (56)^f^									
	A lot	7 (10)^e^		10 (14)^f^									
**Video 3—after watching this video, I feel I learned...**							1.4 (2)		.49		0.101		.49
	Nothing to a small amount	37 (54)^e^		44 (63)^f^									
	A medium amount	25 (36)^e^		19 (27)^f^									
	A lot	7 (10)^e^		7 (10)^f^									
**In general, I found that the information and strategies presented in the resource library were useful to me.**							1.9 (2)		.39		0.120		.39
	Strongly disagree to somewhat agree	30 (48)^g^		28 (42)^h^									
	Agree	28 (44)^g^		30 (45)^h^									
	Strongly agree	4 (6)^g^		9 (13)^h^									
**How much of the different material in the resource library did you actually use?**							3.1 (1)		.45		0.153		.45
	None of it to very little	28 (44)^g^		20 (30)^h^									
	Some, most, or all	35 (56)^g^		47 (70)^h^									

^a^SCOOP: Stress and Coping Online Outreach Program.

^b^n=72.

^c^n=76.

^d^n=71.

^e^n=69.

^f^n=70.

^g^n=63.

^h^n=67.

#### Objective 2

**Figure 3:**
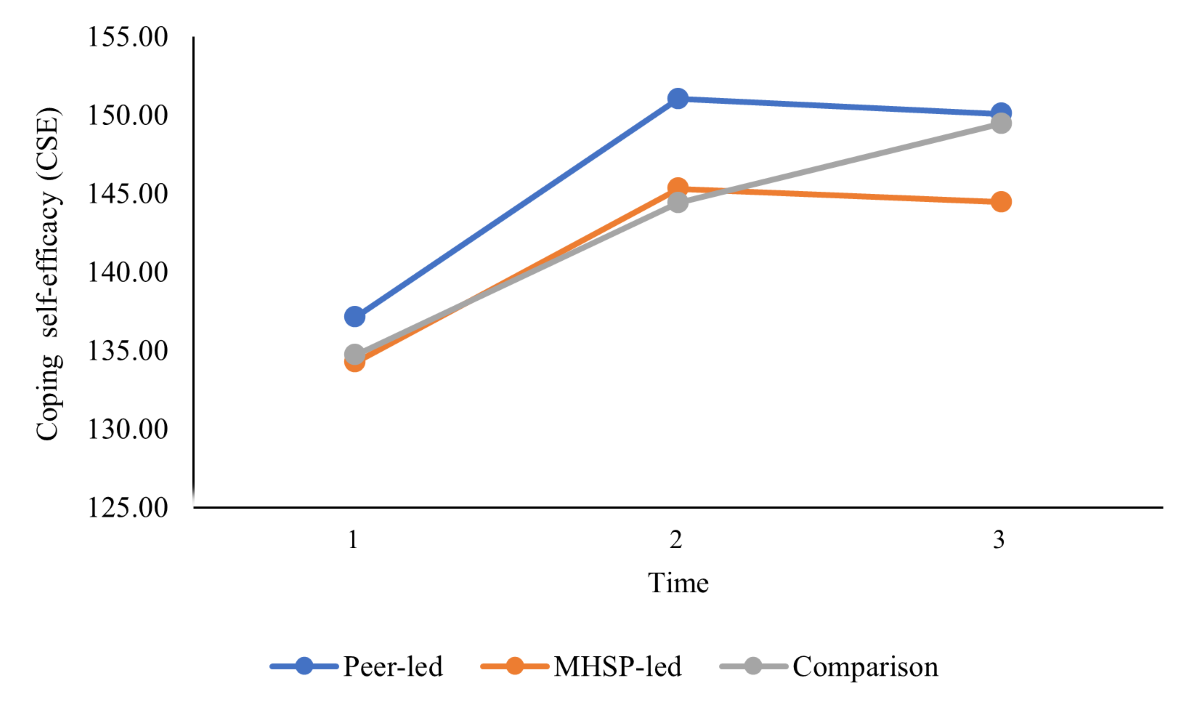
University students’ reported coping self-efficacy over time. Main effect of time represents a significant difference between time 1 and time 2 as well as between time 1 and time 3. CSE: Coping Self-Efficacy Scale; MHSP: mental health service provider.

**Figure 4:**
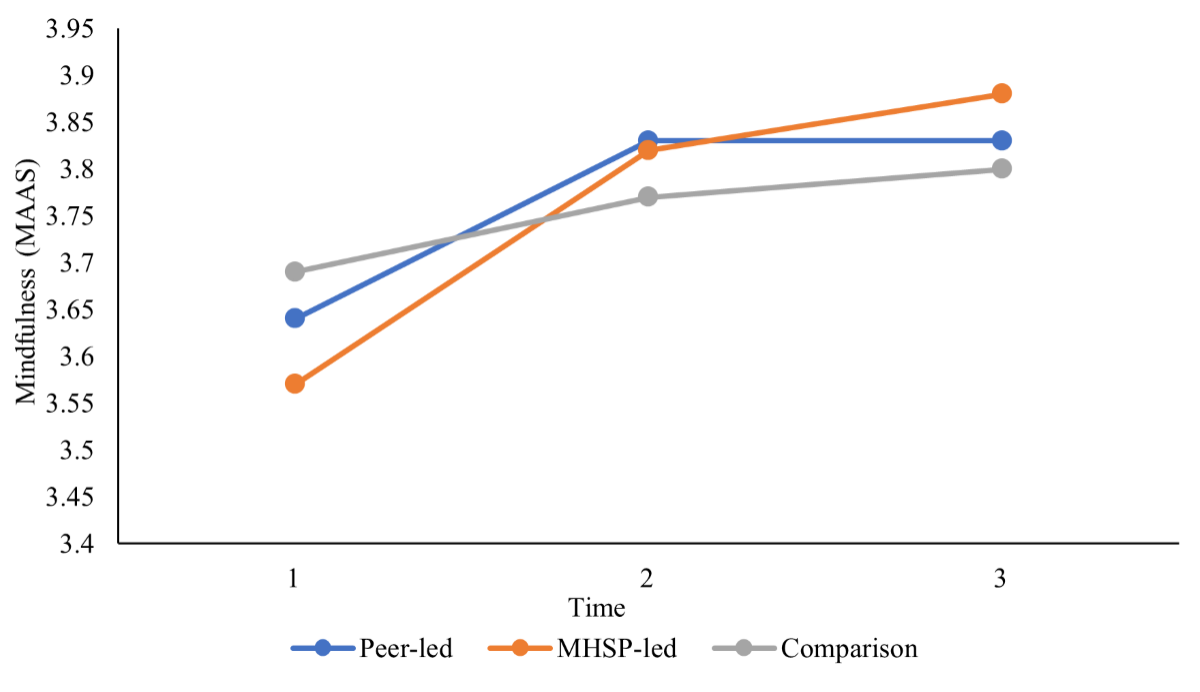
University students’ reported mindfulness over time. Main effect of time represents a significant difference between time 1 and time 2 as well as between time 1 and time 3. MAAS: Mindful Attention Awareness Scale; MHSP: mental health service provider.

**Figure 5:**
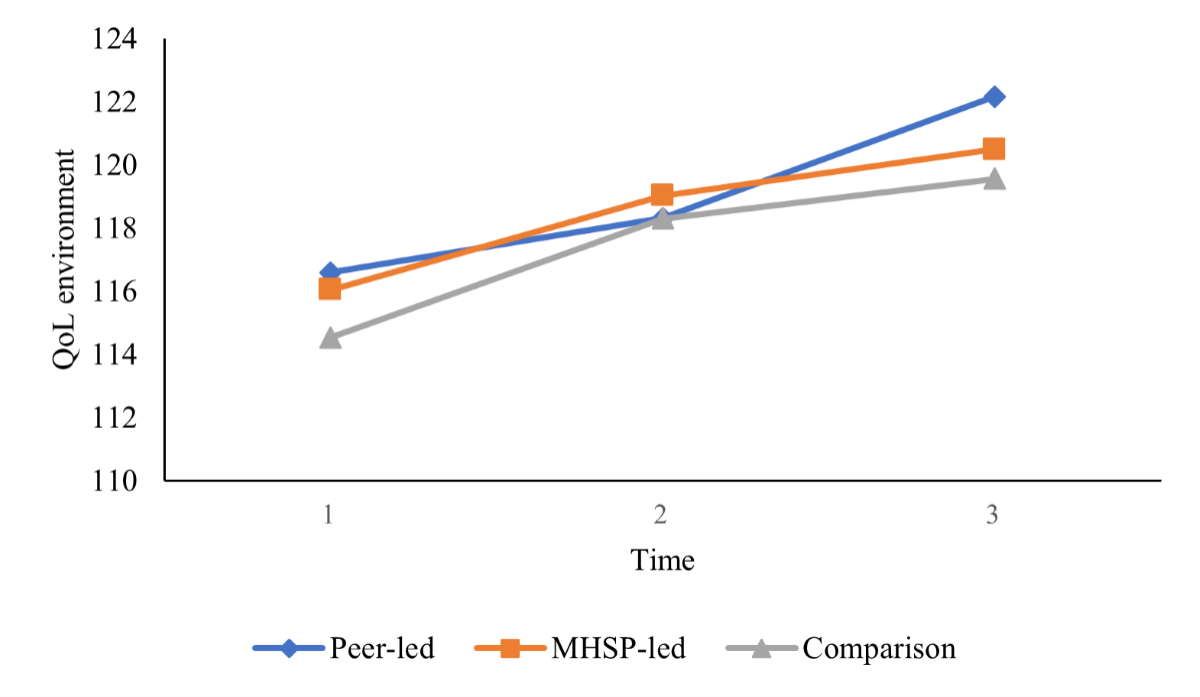
University students’ reported quality of life (QoL; environment) over time. Main effect of time represents a significant difference between time 1 and time 3. MHSP: mental health service provider.

**Figure 6:**
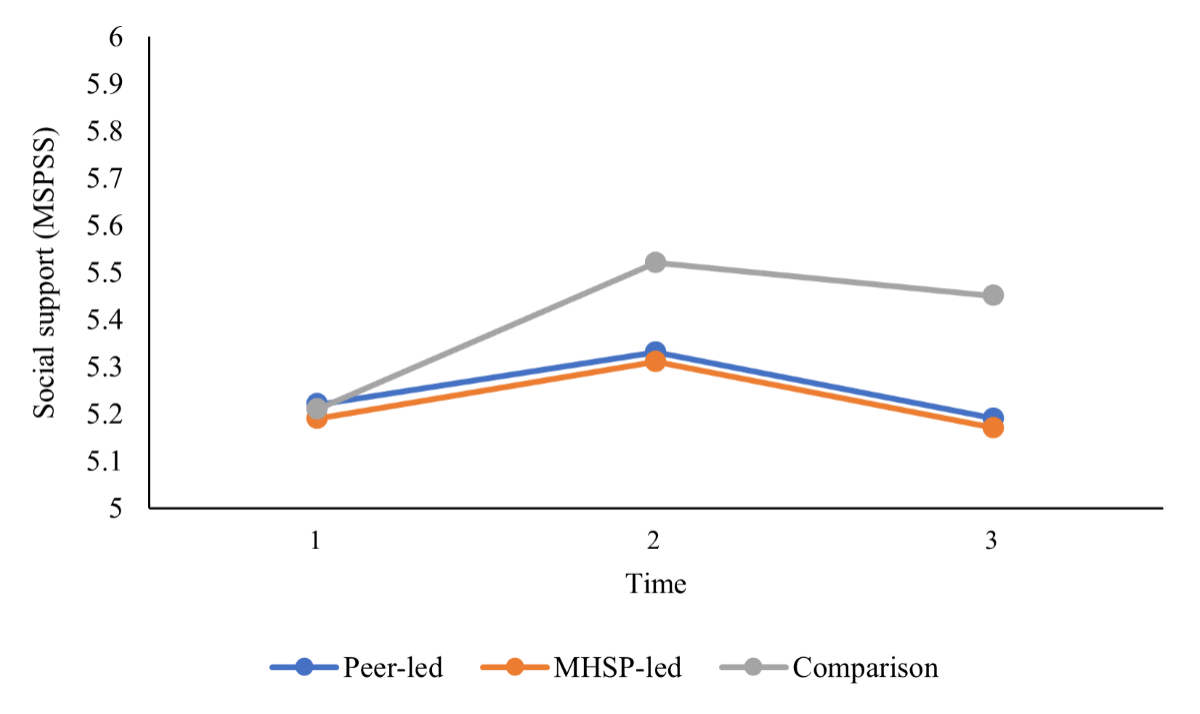
University students’ reported social support over time. Main effect of time represents a significant difference between time 1 and time 2 as well as between time 2 and time 3. MHSP: mental health service provider; MSPSS: Multidimensional Scale of Perceived Social Support.

**Figure 7:**
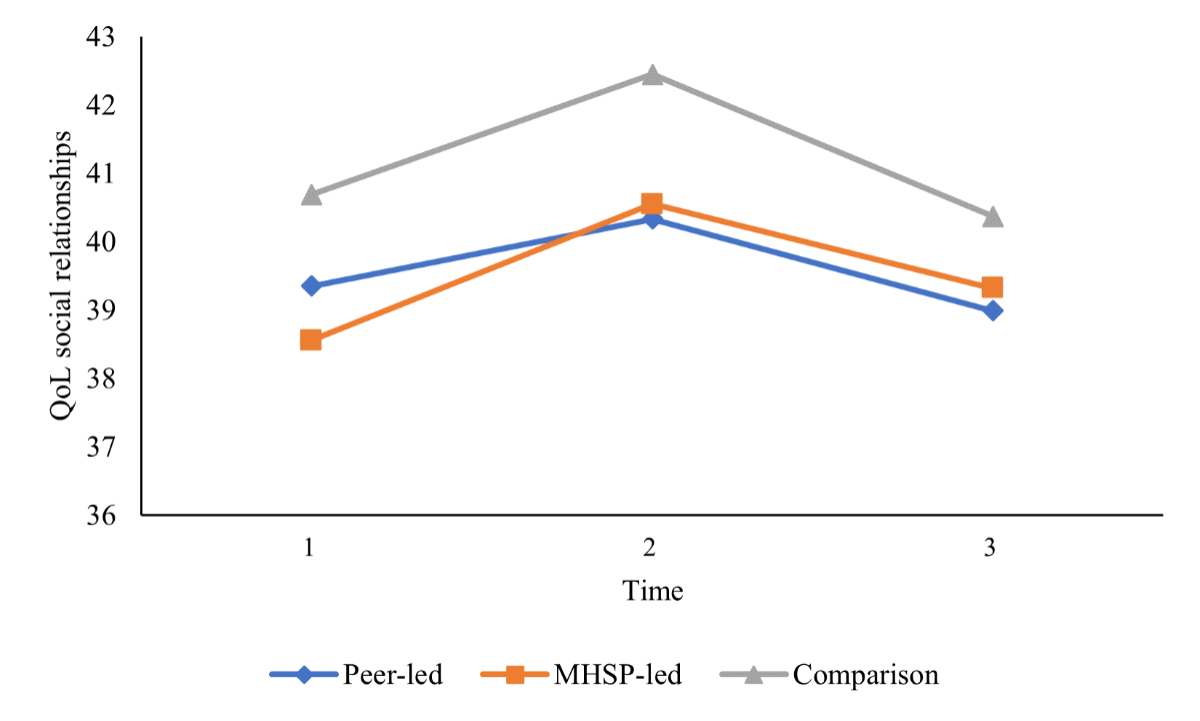
University students’ reported quality of life (QoL; social relationships) over time. Main effect of time represents a significant difference between time 1 and time 2 as well as between time 2 and time 3. MHSP: mental health service provider.

The second objective sought to compare group differences between a mental health service provider–presented versus peer-presented web-based skill-building video outreach program and a wait-list comparison group in terms of well-being outcomes (ie, decreased stress and increased coping self-efficacy, social support, social connectedness, mindfulness, and QoL) at 3 different time points using a series of 2-way mixed ANOVAs. On the basis of the results from the Mauchly test of sphericity indicating that the assumption of sphericity was violated for some of the 2-way mixed ANOVAs, the Greenhouse-Geisser correction was used for all 2-way mixed ANOVAs for a more conservative approach. As presented in Table 4, the results did not reveal any significant 2-way interaction between group (mental health service provider–presented, peer-presented, or wait-list comparison) and time (baseline, after the intervention, and follow-up) on stress, coping self-efficacy, social support, social connectedness, mindfulness, and QoL, indicating that there was no effect of intervention group on any of the well-being outcomes over time. In addition, the results showed that there was no main effect of group for any of the outcomes assessed, which indicates that, regardless of time, there were no group differences on any of the well-being outcomes. However, as reported in Table 4, the main effect of time was statistically significant for coping self-efficacy, social support, mindfulness, the QoL social relationships domain, and the QoL environment domain, which indicates that, overall, regardless of group (mental health service provider–presented, peer-presented, or wait-list comparison), there was a change in these well-being outcomes over time. Pairwise comparisons were conducted using the Bonferroni correction set at a *P* value of .02 (.05 divided by 3 to account for the 3 comparisons used in the analysis) to assess between which time points the time effects occurred. As illustrated in Figures 3 and 4, the results showed that all students increased in coping self-efficacy and mindfulness from time 1 to time 2 and then remained stable at time 3. As illustrated in Figure 5, the results also showed that students increased in the QoL environment domain from time 1 to time 3, although time 2 was not statistically significant with any other time point. Finally, the results of the pairwise comparisons showed that both social support and the QoL social relationships domain significantly increased from time 1 to time 2 and then significantly decreased from time 2 to time 3 (Figures 6 and 7).

**Table 4:** Results of 3 (group: mental health service provider–presented, peer-presented, or wait-list comparison) × 3 (time: baseline, after the intervention, and follow-up) 2-way mixed ANOVAs on well-being outcomes.

Well-being outcome and measurement		*F* test (*df*)	*P* value	η_p_^2^	1 – *β*	Chi-square (*df*)	*P* value
**Stress**							
	Mauchly test of sphericity—time	N/A^a^	N/A	N/A	N/A	17.8 (2)	<.001
	Interaction—Greenhouse-Geisser	1.55 (3.70,396.26)	.19	0.014	.46	N/A	N/A
	Main effect of group (between)	1.03 (2,214)	.36	0.010	.23	N/A	N/A
	Main effect of time (within)—Greenhouse-Geisser	3.70 (1.85,396.26)	.03	0.013	.68	N/A	N/A
**Coping self-efficacy**							
	Mauchly test of sphericity—time	N/A	N/A	N/A	N/A	1.5 (2)	.48
	Interaction—Greenhouse-Geisser	0.61 (3.97,423.08)	.70	0.006	.20	N/A	N/A
	Main effect of group (between)	0.40 (2,213)	.67	0.004	.11	N/A	N/A
	Main effect of time (within)—Greenhouse-Geisser	24.52 (1.99,423.08)	*<*.001	0.103	1	N/A	N/A
**Social support**							
	Mauchly test of sphericity—time	N/A	N/A	N/A	N/A	18.3 (2)	.001
	Interaction—Greenhouse-Geisser	1.94 (3.69,396.52)	.11	0.018	.56	N/A	N/A
	Main effect of group (between)	0.70 (2,211)	.50	0.007	.17	N/A	N/A
	Main effect of time (within)—Greenhouse-Geisser	7.04 (1.85,396.56)	.001	0.032	.93	N/A	N/A
**Social connectedness**							
	Mauchly test of sphericity—time	N/A	N/A	N/A	N/A	11.3 (2)	.004
	Interaction—Greenhouse-Geisser	1.43 (3.80,399.07)	.22	0.013	.43	N/A	N/A
	Main effect of group (between)	2.11 (2,210)	.12	0.020	.43	N/A	N/A
	Main effect of time (within)—Greenhouse-Geisser	1.74 (1.90,399.07)	.18	0.008	.36	N/A	N/A
**Mindfulness**							
	Mauchly test of sphericity—time	N/A	N/A	N/A	N/A	8.0 (2)	.02
	Interaction—Greenhouse-Geisser	0.88 (3.86,408.89)	.48	0.008	.28	N/A	N/A
	Main effect of group (between)	0.14 (2,214)	.99	<0.001	.05	N/A	N/A
	Main effect of time (within)—Greenhouse-Geisser	9.66 (1.93,408.89)	*<*.001	0.044	.98	N/A	N/A
**QoL^b^ (physical health)**							
	Mauchly test of sphericity—time	N/A	N/A	N/A	N/A	1.5 (2)	.48
	Interaction—Greenhouse-Geisser	0.67 (3.97,417.05)	.62	0.006	.21	N/A	N/A
	Main effect of group (between)	0.35 (2,210)	.96	<0.001	.06	N/A	N/A
	Main effect of time (within)—Greenhouse-Geisser	0.63 (1.99,417.05)	.53	0.003	.16	N/A	N/A
**QoL (psychological health)**							
	Mauchly test of sphericity—time	N/A	N/A	N/A	N/A	0.8 (2)	.69
	Interaction—Greenhouse-Geisser	0.23 (3.99,420.49)	.93	0.002	.10	N/A	N/A
	Main effect of group (between)	0.63 (2,211)	.53	0.006	.15	N/A	N/A
	Main effect of time (within)—Greenhouse-Geisser	0.65 (1.99,420.49)	.53	0.003	.16	N/A	N/A
**QoL (social relationships)**							
	Mauchly test of sphericity—time	N/A	N/A	N/A	N/A	11.5 (2)	.003
	Interaction—Greenhouse-Geisser	0.29 (3.80,398.68)	.87	0.003	.11	N/A	N/A
	Main effect of group (between)	0.86 (2,210)	.42	0.008	.20	N/A	N/A
	Main effect of time (within)—Greenhouse-Geisser	4.57 (1.90,398.68)	.01	0.021	.76	N/A	N/A
**QoL (environment)**							
	Mauchly test of sphericity—time	N/A	N/A	N/A	N/A	6.6 (2)	.03
	Interaction—Greenhouse-Geisser	0.27 (3.88,405.37)	.90	0.003	.11	N/A	N/A
	Main effect of group (between)	0.12 (2,209)	.89	0.001	.07	N/A	N/A
	Main effect of time (within)—Greenhouse-Geisser	7.89 (1.94,405.73)	*<*.001	0.036	.95	N/A	N/A

^a^N/A: not applicable.

^b^QoL: quality of life.

## Discussion

### Principal Findings

The overarching goal of this study was to evaluate a mental health service provider–presented versus peer-presented mental health resilience skill–building web-based video outreach program against a wait-list comparison group. Specifically, the first objective sought to compare group differences on the web-based outreach program’s acceptability between the types of deliverers (mental health service provider vs peer). Building on this, the second objective sought to compare group differences between the intervention groups (mental health service provider–presented and peer-presented) and a wait-list comparison group in terms of well-being outcomes (ie, decreased stress and increased coping self-efficacy, social support, social connectedness, mindfulness, and QoL) over 10 weeks.

Interestingly, the web-based outreach program received similarly high acceptability and satisfaction ratings regardless of whether the program deliverer was a mental health service provider or a peer. In both the mental health service provider–presented and peer-presented programs, most participants (58/72, 81% in the mental health service provider–presented group and 69/76, 91% in the peer-presented group) indicated that they were planning to use the program strategies in the future from *sometimes* to *frequently*. In addition, a large proportion of students in the mental health service provider–presented group (46/71, 65%) and in the peer-presented group (53/76, 70%) indicated that they felt that, after watching video 1, they had learned *a medium amount* to *a lot.* Thus, acknowledging the need to integrate cost-effective and easily accessible mental health programs to build mental health resilience capacity and support students in coping with general stress, these findings provide promising early evidence that a web-based skill-building resource for teaching mental health resilience skills is satisfactory and acceptable for university students. Furthermore, this may be a particularly valuable approach for students who have a preference for peer-led approaches or to complement professional mental health services. This is in line with previous literature reporting high satisfaction with web-based mental health skill-building programs [8,60]. However, to our knowledge, this is the first study to examine such acceptability of and satisfaction with a peer-presented versus mental health service provider–presented universal resilience-building web-based program in a sample of university students. Interestingly, given nonsignificant differences between the groups, the findings suggest that a resilience skill–building video outreach program may be acceptable for university students regardless of service delivery type (mental health service provider–presented or peer-presented).

Although most students reported high overall acceptability and satisfaction with the program, a certain proportion of students (eg, students saying that they were never to rarely planning to use the strategies in the future: 21/148, 14.2%) did indicate less-positive reports regarding the program. However, it is important to note that a random convenience sample was recruited, where students were generously compensated for their time, to avoid a self-selection bias; therefore, a broad sample of students who may or may not have had the need for or interest in these strategies participated in the study. Thus, it is not surprising that a number of the participants reported less interest in the program or willingness to use the strategies in the future as they may not have had a need for these strategies. These findings further confirm that mental health outreach will usually be of interest and relevance specifically to those who are currently feeling a need for this support.

Moreover, interpretation of this study’s findings needs to be carried out with a particular focus on the societal context in which the program was delivered. Importantly, while the program was being delivered, a state of emergency because of the COVID-19 pandemic was declared in the province in which this study was conducted. This brought on significant changes for students, such as social distancing restrictions and changes related to remote learning. Thus, such elevated levels of reported acceptability and satisfaction are encouraging as the program was disseminated at the beginning of the COVID-19 pandemic, when there was much uncertainty and students’ lifestyles were rapidly changing [44,61]. In addition, the elevated proportion of students who reported having used the program strategies to cope with COVID-19 stress suggests that these types of strategies are feasible to use in times of rapid change and uncertainty. However, considering the societal context, this may have played a role in the nonsignificant group differences between the satisfaction with the mental health service provider–presented program and the satisfaction with the peer-presented program. Given the challenges associated with the pandemic, students may have been eager to access web-based mental health resources regardless of who was delivering the program. Although the findings may have important implications for the development and integration of future outreach programs seeing this program’s high acceptability and satisfaction, nonsignificant group differences should be interpreted with caution based on the context, and further investigation may be required.

The second objective was to compare group differences between a mental health service provider–presented versus peer-presented web-based skill-building video outreach program and a wait-list comparison group in terms of well-being outcomes over time (baseline, after the intervention, and follow-up). Although the students rated the program very positively, no difference was found among any of the 3 groups on any of the well-being outcomes over time. Thus, the intervention groups did not demonstrate a greater improvement over time in well-being outcomes relative to the wait-list comparison group, although, as discussed below, there was a general increase in well-being for all groups. Even though previous studies have found that web-based interventions are effective in supporting university students’ well-being [62], there are several potential factors that may explain this lack of a detectable intervention benefit. It may be that this null finding indicates that the intervention was ineffective, meaning that perhaps the intervention was not optimized (eg, the time span was too short or the students did not engage with the strategies for a sufficient amount of time). As demonstrated in the literature, intervention dosage is crucial to an intervention [63]; as such, it may be that students need supplemental time to engage with the strategies and for strategy practice.

An alternative hypothesis for this null finding is that all 3 groups of participants may have had increased access to mental health support resources through the university and community given the plethora of web-based student mental health resources offered because of the pandemic. The elevated reports of stress associated with the COVID-19 pandemic and the need to move students to web-based learning resulted in a plethora of web-based resources offered through the university and community to effectively support students during this time. In summary, all 3 groups in this study would have had access to several mental health support resources through the university and community as well as a decrease in academic stressors.

Although no group differences were found, there was a significant change over time in coping self-efficacy, social support, mindfulness, and QoL (social relationships and environment domains) for all 3 groups. Specifically, students increased in coping self-efficacy and mindfulness from time 1 to time 2 and remained stable from time 2 to time 3. Similarly, students reported an increase in the quality of their environment (ie, QoL environment domain) between time 1 and time 3. These findings are in line with a study conducted by Hamza et al [64] demonstrating that students with preexisting mental health concerns reported an increase or similar levels of psychological well-being compared with a year before. Similar to the findings of Hamza et al [64], many students in this sample were already reporting mental health difficulties and may have been better able to cope with the changes associated with the COVID-19 pandemic, such as increased social isolation.

Moreover, mindfulness practice has been gaining popularity as an evidence-based strategy for managing stress. As a result, several means of support offered to deal with the pandemic stress were aimed at enhancing mindfulness [65,66], which may have influenced students’ reported mindfulness during this time. Finally, the increase in the QoL environment domain has been hypothesized to have been affected by potential positive experiences of the COVID-19 pandemic, as noted in several recent COVID-19 studies [67,68]. The QoL environment domain measures facets such as “Opportunities for acquiring new information and skills” and “Participation in and opportunities for recreation” and may have increased as a result of the reduction of academic stressors and increase in time available for leisure activities.

Interestingly, the pattern of change for social support and the QoL social relationships domain was different, with an increase from time 1 to time 2 before returning to baseline levels at time 3. The increase in social support and the QoL social relationships domain is consistent with previous literature on natural or societal disasters, where there is an increase in social and general mental health support directly following these tragic events [69,70]. Thus, the time 2 increase in perceived social support is hypothesized to have been related to the early pandemic surge in social connections as families, peers, and communities reached out to individuals to ensure safety and well-being. This early pandemic increase in social support is similar to findings from a community sample of adults, where an increase in social support was reported at the start of the pandemic [71]. However, although interesting patterns emerged in terms of students’ well-being during this time, the findings of this study are tentative, as several factors may have affected the results, and should be interpreted with caution.

### Limitations and Future Directions

This study is not without limitations. Considering that the program was disseminated during a time of change because of the onset of the COVID-19 pandemic, the results may have been different if the web-based outreach program had been provided to manage regular day-to-day stress. Thus, these results may not be generalizable to nonpandemic times, and future research would benefit from evaluating program effectiveness in a different context. Furthermore, given the importance of intervention dosage [63], future studies may want to examine specifically how much time students engage with strategy practice. Although this study asked students to retrospectively rate on a Likert scale from *never* to *frequently* how much they used the program strategies, it may be helpful to have students provide a daily report of the time spent engaging with each strategy to be able to better evaluate whether the dosage had an impact on the effectiveness of the intervention. A further limitation of this study is the use of a nonstandardized, researcher-designed measure to assess program satisfaction owing to the lack of relevant standardized measures in this area. Finally, there is limited generalizability of the findings owing to a sample where the participants predominantly identified as White women. Further research is needed to explore student responses to web-based mental health outreach programs among groups that may be underrepresented in our sample.

### Conclusions

The elevated levels of mental health distress reported by university students and the difficulties associated with the developmental period of emerging adulthood highlight the need to provide university students with appropriate mental health support. Although this study did not demonstrate program effectiveness, students reported high acceptability of both mental health service provider–presented and peer-presented programs. The findings highlight that the content presented (strategies for skill-building and psychoeducation) may play a more important role in students’ acceptability than who is delivering the program. Hence, future initiatives may want to consider the involvement of peers in delivering similar web-based programs as an effective approach to address barriers to program dissemination, such as limited resources and increasing acceptability of students with a preference for peer approaches.

## Appendix

Multimedia Appendix 1 CONSORT-eHEALTH checklist (V 1.6.1).

## Abbreviations

CSE: Coping Self-Efficacy Scale
GHSQ: General Help-Seeking Questionnaire
MAAS: Mindful Attention Awareness Scale
MSPSS: Multidimensional Scale of Perceived Social Support
PSS: Perceived Stress Scale
QoL: quality of life
SCS-R: Social Connectedness Scale-Revised
WHOQOL-BREF: World Health Organization Quality of Life Brief questionnaire
